# Factors influencing parents’ hesitancy to vaccinate their children aged 5–11 years old against COVID-19: results from a cross-sectional study in Malaysia

**DOI:** 10.3389/fpubh.2023.1091015

**Published:** 2023-05-16

**Authors:** Roy Rillera Marzo, Ritankar Chakraborty, Shean Yih Soh, Hui Zhu Thew, Collins Chong, Ching Sin Siau, Khairuddin Bin Abdul Wahab, Indang Ariati Binti Ariffin, Shekhar Chauhan, Ken Brackstone, Bijaya Kumar Padhi, Petra Heidler

**Affiliations:** ^1^Department of Community Medicine, International Medical School, Management and Science University, Selangor, Malaysia; ^2^Global Public Health, Jeffrey Cheah School of Medicine and Health Sciences, Monash University Malaysia, Bandar Sunway, Malaysia; ^3^International Institute for Population Sciences (IIPS), Mumbai, India; ^4^Department of Psychiatry, Faculty of Medicine and Health Sciences, Putra Malaysia University, Serdang, Malaysia; ^5^Department of Family Medicine, Faculty of Medicine and Health Sciences, Putra Malaysia University, Serdang, Malaysia; ^6^Department of Anaesthesia and Intensive Care, Faculty of Medicine and Health Sciences, Universiti Putra Malaysia, Serdang, Malaysia; ^7^Centre for Community Health Studies, Faculty of Health Sciences, Universiti Kebangsaan Malaysia, Kuala Lumpur, Malaysia; ^8^Clinical Informatics Research Unit, Faculty of Medicine, University of Southampton, Southampton, United Kingdom; ^9^Asian Institute of Public Health, Bhubaneswar, India; ^10^Department of Community Medicine and School of Public Health, Post Graduate Institute of Medical Education and Research (PGIMER), Chandigarh, India; ^11^Institute International Trade and Sustainable Economy, IMC University of Applied Sciences Krems, Krems an der Donau, Austria; ^12^Department of Health Sciences, St. Pölten University of Applied Sciences, St. Pölten, Austria

**Keywords:** vaccine hesitancy, COVID-19, children, Malaysia, health education and awareness

## Abstract

**Introduction:**

Vaccination programs have been rolled out across the globe to contain and mitigate the spread of the COVID-19 infection. Until recently, such programs were limited to adults and the older population, thereby limiting children from getting vaccinated. Recently, the Malaysian government rolled out vaccination for children aged 5–11 years. However, there are certain factors that might affect vaccination uptake among children. This study explores factors influencing parents’ hesitancy to vaccinate children in Malaysia.

**Method:**

A nationwide online cross-sectional convenience sampling survey from April 21, 2022 to June 3, 2022 was conducted. The study used descriptive statistics to inform about vaccine hesitancy among parents. Cross-tabulation was performed to calculate the frequency and percentage of vaccine hesitancy, quality of life, e-health literacy, and the 5C psychological antecedents of vaccination among parents with children 5-11 years in Malaysia. Graphical methods were used to portray the levels of e-health literacy and levels of 5C psychological antecedents of vaccination. The study used both bi-variate and multivariate analysis to understand the relationship between vaccine hesitancy and the socio-demo-economic factors, quality of life, e-health literacy and 5C psychological antecedents.

**Results:**

Of 382 participants, almost one-third (33%) of participants reported vaccine hesitancy for their children. For 5C’s psychological antecedents of vaccination, around one quarter (26.96%) reported disagreement for confidence in vaccination, almost half (52.36%) reported disagreement for vaccination complacency, three-fifths (60.99%) reported vaccination constraint, one quarter (25.92%) reported calculation antecedent, and almost one-third reported disagreement over collective responsibility antecedent (25.92%). Chi-square test revealed that gender, employment status, and parents’ COVID-19 vaccination status were significantly associated (*p*<0.05) with vaccine hesitancy among parents. Assessing the influence of transactional e-health literacy, only the communication component contained a significant association (*p*<0.05). Among the 5C psychological antecedents, confidence, calculation, and collective responsibility were significantly associated (*p*<0.05) with vaccine hesitancy. Parents with secondary [OR: 8.80; CI: 2.44−31.79, (*p*<0.05)], post-secondary [OR: 5.21; CI: 2.10-13.41, (*p*<0.05)], and tertiary education [OR: 6.77; CI: 2.25−20.35, (*p*<0.05)] had significantly higher likelihood of vaccine hesitancy than those with primary education.

**Conclusion:**

Highly educated parents are more skeptical and are more likely to perceive the vaccine as unsafe and ineffective for their children. It is critical to disseminate the required information about the vaccine safety to the educated group.

## Introduction

The novel coronavirus disease (COVID-19) was first isolated from the Chinese city of Wuhan in December 2019, and has been the source of the most recent global health crisis (1–3). Malaysia detected its first three cases of COVID-19 before the declaration of pandemic status on 25 January 2020 (4–7). Since then, Malaysia has undergone multiple waves of COVID-19 infections. As of 5 October 2022, there were 4.85 million of confirmed COVID-19 cases in Malaysia, while there were 619 million cases globally at the same time (1, 2). Vaccination programs have been since rolled out across the globe to contain and mitigate the spread of the COVID-19 infection. The Malaysian government pledged its commitment toward the COVID-19 Vaccine Global Access (COVAX) partnership, which the World Health Organization (WHO) spearheaded (5). The administration of COVID-19 vaccines in Malaysia was administered via the National COVID-19 Vaccine Immunization Program (Program Imunisasi COVID-19 Kebangsaan – PICK), which began on 24 February 2021 (8)– (9). There were three vaccines first introduced to the country vaccination program, namely AZD1222 (AstraZeneca), BNT162b2 (Pfizer-BioNTech), and CoronaVac (Sinovac) (8, 9). On 23 September 2021, Malaysia began offering COVID-19 vaccines to adolescents in the country, primarily employing the BNT162b2 vaccine (10, 11). It was not until February 2022, however, that the same BNT162b2 vaccine was offered to children aged 5–11, while CoronaVac was too provided to this group in March 2022 (12).

Clinical evidence has shown the efficacies of these vaccines against COVID-19-related mortality, hospitalization, and symptomatic illness (8). Despite this, a small number of people may be unsure about the acceptance of certain vaccines. Vaccine hesitancy was defined by the Strategic Advisory Group of Experts on Immunization (SAGE) as “the delay in acceptance or refusal of vaccination despite the availability of vaccine services” (13, 14), and is regarded a certain hindrance for a country to achieve herd immunity against COVID-19.

A recent systematic review explored vaccine acceptance rates across the globe and found that Malaysia had the highest acceptance rate (94.3%) for COVID-19 (13, 15). Various studies have also explored the attitude of the Malaysian population toward COVID-19 vaccines and the phenomenon of vaccine hesitancy among adults (16, 17). However, these studies primarily focused on vaccine hesitancy rates among adults. A recently published study investigated parents’ willingness to vaccinate their children under 12 years old against COVID-19, and 73.6% of their participants were willing to vaccinate their children (18). Parental levels of vaccine hesitancy, together with parental knowledge and attitudes about the disease and vaccine, has been shown to predict willingness to vaccinate their children (3, 4).

Examining coronavirus-related issues has remained elusive in the Malaysian context as far as vaccine hesitancy for children is concerned, especially from the parents’ perspective. To our knowledge, there is no published study investigating the willingness of parents to vaccinate their adolescent children at the time of writing. However, authors found some studies pertaining to health literacy related to COVID-19 vaccination, although these studies did not examine health literacy from parent’s perspective as we intend to examine in this study (19, 20). Some countries have conducted surveys to investigate COVID-19 vaccine hesitancy among parents with children aged 5–11 years old. In the United States, 40.75% of Malaysian parents were willing to vaccinate their children (21). In research involving multiple Middle Eastern countries, 32% of children were vaccinated against COVID-19 (22). A quarter of mothers participating in a survey in Saudi Arabia expressed hesitancy about their children’s vaccination (23). There were also varying degrees of parental vaccine hesitancy among the Asian regions. Only 11.7–19.4% of parents expressed hesitancy in the Chinese cities of Shandong and Zhejiang (24), whereas 35.3% of Japanese parents were hesitant to vaccinate their children (25). In contrast, more than 70% of the parents in Hong Kong were hesitant to vaccinate their children (26). Some studies have applied health beliefs and planned behavior theoretical models (27–29) to examine the relationship between vaccine hesitancy and parental attitudes and beliefs.

The most consistent predictors of parents’ COVID-19 vaccine resistance for children 5–11 years are a lack of confidence in the safety and effectiveness of the vaccine, followed by lack of trust in government, perceptions that children are not susceptible to the disease, and a lack of community and family support for vaccinating children against COVID-19 (29–42). Positive attitudes toward vaccination experiences or outcomes may also play a role in predicting parental willingness to vaccinate their children (29). Demographic variables have also been associated with parental COVID-19 vaccine acceptance. These include higher parental income and education, and whether the parent has received the COVID-19 vaccination themselves (30, 32, 35). Racial and ethnic differences have also been reported. For example, Asian-American parents were most likely to vaccinate their 5–11-year-old and 12–17-year-old children, whereas non-Hispanic White parents were least likely (43, 44). These differences in parental intentions appear to coincide with the race and ethnicity of older children who have been vaccinated based on their share of the population (45).

The advancement of technology has a major influence on how health information is disseminated and received by the general public. The Transactional Model of eHealth Literacy was first proposed to define a hierarchy of skillsets that may have an impact on an individual’s healthcare engagement experience (46). For example, functional eHealth literacy is defined as basic skills in writing and reading about health on the internet; communicative eHealth literacy involves skills that allowed communication about health with other users on the online environment; and critical eHealth literacy refers to the ability to evaluate the information obtained and understand the risk in the sharing of such information. The ability to apply health knowledge obtained via the internet across different setting forms the highest cognitive level of eHealth literacy and this is termed translational eHealth literacy (46). Health literacy has been proposed as an important element in the fight against COVID-19 and higher health literacy has been associated with higher likelihood of intention to be immunized (47–49).

Further, the 5C model of psychological antecedents, which include confidence, complacency, constraint, calculation, and collective responsibility, is often used to predict vaccination hesitancy in a population (50, 51). Generally, people with greater confidence trust the safety and efficacy of COVID-19 vaccines, and also believe in the competence and reliability of their local healthcare service providers in delivering the vaccination program (32). On the contrary, people lacking confidence may adopt a conspiracy mentality with regards to vaccines, and worry about the harmful effects of vaccine (50). Complacency in the 5C model of psychological antecedents was defined as having low perceived risk of the vaccine-preventable disease and eventually deeming vaccination as unnecessary (50). In view of this, complacent people may express lower intention to receive vaccination. Constraints may be seen as barriers that impede the vaccination process. These barriers may include the accessibility, availability, and affordability of vaccines and the willingness-to-pay of the population (50). Calculation in the 5C model refers to people who would undergo extensive information gathering. Depending on the sources of information search, calculation may influence vaccination attitudes in diverse ways. For example, if anti-vaccination or vaccine - critical information was found, calculation would potentially lead to higher hesitancy (50).

Herd immunity is an important public health concept that can be used to mitigate the spread of an infectious disease. Consequently, some may be willing to receive vaccination in order to protect others within the community. This is termed collective responsibility (50).

Our study aimed to investigate Malaysian parents’ hesitancy, health literacy, and behaviors surrounding COVID-19 vaccinations of their children aged 5–11 years old, and to examine the psychological barriers that may prevent parents in Malaysia from getting their children vaccinated against the COVID-19 virus.

## Method

### Study design, respondents, and sample size

We conducted a nationwide online cross-sectional study in Malaysia from April 21, 2022 to June 3, 2022. The inclusion criteria of the study were as follows: parents with children 5–11 years, and currently a resident in Malaysia. Participants who were unable to read English, those not given consent, and non-citizens of Malaysia were excluded from the study. To calculate the required sample size for a single proportion, we used Pocock’s formula: *n* = *Zα* 2 *p*(1-*p*)/*d*2, where *n* = minimum required sample size, (Zα) = 1.96, d (precision) = 5%, and P = expected prevalence. Based on our extensive literature search, the prevalence of vaccine hesitancy was found to be within the range of 10 to 30%. Using these percentages, an estimated sample size of between 138 and 318 was required. The average provided a required minimum sample size of 227. Considering potential dropouts and incomplete forms (227 + [20%]), 275 was our target sample size. A total of 1,000 questionnaires were distributed with 38.2% response rate.

### Procedure

A pilot study was conducted prior to the actual data collection to test the survey questions, assess the feasibility of the study design, and identify any potential issues. An online survey was used for data collection because of the ongoing COVID-19 pandemic in the country. Participants were recruited using convenience sampling method. Questionnaires were disseminated via various social media platforms such as Instagram, Twitter, Facebook, WhatsApp, and Telegram. Participants completed an online consent form after confirming that they understood the purpose, risks, and benefits of the study. Time for completion of questionnaire was approximately 10–15 min. No incentive was offered for completing the questionnaire. We conducted a follow-up and reminder to ensure a higher response rate. Specifically, we sent a reminder email to all participants who had not completed the survey after 1 week. Face and content validity were considered satisfying by the multidisciplinary panel of five experts including a psychiatrist, clinical psychologist, physician, pharmacist, and public health expert for critical review, content, and face validity. All items were evaluated for necessity, clarity, and relevance. The content validity and face validity were 88 and 92%, respectively.

### Measures

#### Demographics

Malaysian participants indicated their age, gender (male, female), residential area (rural, urban), educational level (no formal education, primary, secondary, tertiary), marital status (single, married, divorced, widowed), employment (unemployed, part-time, full-time), religion (Christian, Buddhism, Muslim, Hinduism, Other, None), ethnicity, and household income.

#### Quality of life

Next, participants were asked about their perceived quality of life (“How would you rate your quality of life?”), which was rated on a 5-point Likert scale (1 = *very poor*; 5 = *very good*; *M* = 4.57, *SD* = 1.48). The higher score indicates better quality of life.

#### Parents’ hesitancy to vaccinate their children

This was measured by a single item asking the participants to rate the extent they felt likely to vaccinate their children with the COVID-19 vaccine on a 6-point Likert scale (1 = *very likely*; 6 *very unlikely*; *M* = 4.57, *SD* = 1.48). Higher scores indicate greater hesitancy. A point greater than or equal to 4 was considered overall likely to vaccinate the child, whereas a point of less than 4 suggested that the parents were overall unlikely to vaccinate their child. So, a score of <4 was coded as 1 and was labelled as “Unlikely,” and a score of 4 and above was coded as 0 and labelled as “Likely” and thus converted into a binary variable.

#### 5C’s psychological antecedents of vaccination

The 5Cs were assessed using the previously validated 5C scale (32). The scale consisted of 15 items. Each of the Cs — Confidence, Constraints, Calculation, Complacency and Collective responsibility — were captured using three items. Responses were provided on a six-point Likert scale (1 = *strongly disagree*; 6 = *strongly agree*). The scale was adapted to focus on COVID-19 vaccinations.

All items were scored in a way such that a higher score indicates a higher degree of the C assessed. Internal consistency, as reflected by Cronbach’s alpha, were acceptable in our sample: Confidence α = 0.87, *M* = 4.57, *SD* = 1.48; Complacency α = 0.75, *M* = 4.57, *SD* = 1.48; Constraints α = 0.88 *M* = 4.57, *SD* = 1.48; Calculation α = 0.76, *M* = 4.57, *SD* = 1.48; Collective responsibility α = 0.77, *M* = 4.57, *SD* = 1.48.

The 5C psychological antecedents of vaccination consisted of 5 domains. Each of the domains had 3 questions which were measured using a 6-point Likert Scale (1 = *strongly disagree*; 6 = *strongly agree*). So, the total possible score of each domain was 18. A cumulative score of greater than 9 was taken as overall agreement to that domain, whereas a score of less than or equal to 9 was taken as overall disagreement to that domain. It was then recoded as 1 “Disagree” and 2 “Agree” and converted to a binary variable. Thus 5 binary variables were created for each domain of the 5C psychological antecedent of vaccination.

#### e-Health Literacy

Participants’ vaccine literacy was captured by the COVID-19 vaccine literacy scale (45). The scale was comprised of four components, including functional, communication, critical, and translational skills. Each component was measured using four items on a 6-point Likert scale (1 = *strongly disagree*; 6 = *strongly agree*; α = 0.86, *M* = 4.57, *SD* = 1.48). The total score ranged from 16 to 48. The higher the score, the higher the COVID-19 vaccine literacy.

### Ethical considerations

The anonymous survey data were confidentially stored with password-protected security standards. We utilized an online informed consent process that required participants to read and agree to the terms of participation before beginning the study. The online consent form provided participants with a clear explanation of the study’s purpose, procedures, risks, benefits, confidentiality, and the participant’s rights as a research subject, including information on the use and anonymization of the data and how survey responses guarantee the anonymity of each participant. Participants were also informed that their participation was voluntary, and they could withdraw at any time without consequence. We ensured that participants could only access the survey once to prevent multiple responses. This study was conducted according to the Declaration of Helsinki. Online informed written consent was obtained from all participants before the commencement of the study. Ethics approval was obtained from the Medical Research and Ethics Committee of the Management and Science University.

### Data analysis

Vaccine hesitancy was taken as the primary outcome variable for this study. The variable was recoded as a binary variable (Yes = 1/No = 0) for the primary analysis. Socio-economic and demographic categorical predictors included gender, marital status, highest qualification level, household monthly income, ethnicity, religion, employment status, residential area, insurance status of the child, parents’ comorbidity status, comorbidity status of child, parents’ COVID-19 vaccination status, oldest child’s (5–11 years) COVID-19 vaccination status, side effects experienced by respondent after getting the COVID-19 vaccination, side effects experienced by the oldest child (5–11 years) after getting the COVID-19 vaccination, and risk perceptions about COVID-19. Quality of life, transactional e-health literacy, and 5C psychological antecedents to vaccination were also categorized based on scores from the Likert scale and included in the regression analysis.

### Statistical analysis

The study used descriptive statistics to assess vaccine hesitancy among parents with children under 18 years old in Malaysia based on each socio-economic and demographic characteristics. Cross-tabulation was performed to calculate the frequency and percentage of vaccine hesitancy, quality of life, e-health literacy and 5C psychological antecedents of vaccination among parents with children 5–11 years in Malaysia. Graphical methods were used to portray the levels of e-health literacy and levels of 5C psychological antecedents of vaccination among the study subjects.

Next, the study used bi-variate and multivariate analysis to understand the relationship between vaccine hesitancy and the socio-demo-economic factors, quality of life, e-health literacy, and 5C psychological antecedents. Chi-square tests were performed to examine the association between vaccine hesitancy and the different predictor variables. *p*-values were reported to understand the significance of the association. Multivariate analysis included both unadjusted and adjusted binary logistic regressions with vaccine hesitancy as the binary outcome variable and the predictors as categorical variables.

The adjusted regression was performed taking into account all the predictor variables in a single model. This was used to understand the effect of a predictor variable on the outcome variable after adjusting for the effect of other predictor variables. Odds Ratios (OR) with 95% Confidence Intervals and *p*-values were reported to understand the significance of the relationship between the outcome variable and the predictor variables. ORs greater than 1 indicated a greater chance of vaccine hesitancy while ORs of less than 1 indicated a lesser risk of vaccine hesitancy.

## Results

Table 1 shows percentages of vaccine hesitancy among parents with children 5–11 years old for socio-economic and demographic characteristics. Of the 382 participants, 33% of respondents expressed hesitation in vaccinating their children. In Table 2, among those hesitant to vaccinate their children, the majority were married couples (68.3%). People with lower household income expressed lower hesitation in vaccinating their children, as were people with post-secondary and tertiary education. Among those who were unlikely to vaccinate their children, a majority (62%) were full-time employees and residing in rural areas (78.6%). Further, 73.0% of the hesitant parents had insurance for their children. Among the 126 respondents who expressed hesitancy in vaccinating their children, 108 (85.7%) were without any chronic illness.

**Table 1:** Percentage of COVID-19 vaccine hesitancy among parents with children 5–11 years old.

Vaccine hesitancy	Frequency	Percentage
No	256	67.02
Yes	126	32.98
Total	382	100

**Table 2:** Vaccine hesitancy among parents with children 5–11 years old.

Vaccine hesitancy →	No (*n*, %)	Yes (*n*, %)
Socio-economic and demographic variables ↓		
Age (Mean = 39.5)		
Gender		
Male	88 (34.0)	58 (46.0)
Female	168 (66.0)	68 (54.0)
Marital Status		
Single	83 (32.4)	25 (19.8)
Married	142 (55.5)	86 (68.3)
Divorced	10 (3.9)	8 (6.4)
Widowed	2 (0.8)	1 (0.8)
Single parent	19 (7.4)	6 (4.8)
Highest qualification level		
Primary	57 (22.3)	16 (12.7)
Secondary	23 (9.0)	13 (10.3)
Post-secondary	99 (38.7)	54 (42.9)
Tertiary education	77 (30.0)	43 (34.1)
Household Income		
Equals/less than RM 4,850	125 (49.0)	61 (48.4)
RM 4,850 to RM 10, 959	102 (40.0)	46 (36.5)
More than RM 10, 960	28 (11.0)	19 (15.1)
Ethnicity		
Malay	92 (36.1)	34 (27.0)
Chinese	46 (18.0)	28 (22.2)
Indian	97 (38.0)	59 (46.8)
Others	20 (7.9)	5 (4.0)
Religion		
Islam	98 (38.3)	39 (31.0)
Christianity	30 (11.7)	17 (13.5)
Buddhism	30 (11.7)	17 (13.5)
Hinduism	88 (34.4)	49 (38.9)
Others	10 (3.9)	4 (3.2)
Employment status		
Employed: full time	140 (54.7)	78 (61.9)
Employed: part time	9 (3.5)	9 (7.1)
Self-employed	19 (7.4)	12 (9.5)
Retired/Pensioners	2 (0.8)	3 (2.38)
Housewife/Househusband	17 (6.6)	10 (7.9)
Unemployed	69 (27.0)	14 (11.1)
Residential area		
Urban	53 (20.7)	27 (21.4)
Rural	203 (79.3)	99 (78.6)
Insurance status of child		
Insured	178 (69.5)	92 (73.0)
Not insured	78 (30.5)	34 (27.0)
Parent’s comorbidity status		
With chronic illness	36 (14.1)	18 (14.3)
Without chronic illness	220 (85.1)	108 (85.7)
Comorbidity status of child		
With chronic illness	19 (7.4)	6 (4.8)
Without chronic illness	237 (92.6)	120 (95.2)
Parent’s COVID 19 vaccination status		
Not vaccinated	3 (1.2)	0 (0.00)
Completed 1 dose	0 (0.0)	2 (1.6)
Completed 2 doses	23 (9.0)	35 (27.8)
Completed more than 2 doses	230 (89.8)	89 (70.6)
Oldest child’s COVID 19 vaccination status		
Not vaccinated	85 (33.3)	54 (42.9)
Completed 1 dose	48 (18.8)	23 (18.2)
Completed 2 doses	122 (47.8)	49 (38.9)
Side effects experienced after getting COVID 19 vaccination		
Yes	90 (35.3)	44 (34.9)
No	165 (64.7)	82 (65.1)
Side effects experienced by oldest child after getting COVID 19 vaccination		
Yes	44 (17.2)	25 (19.8)
No	211 (82.8)	101 (80.2)
Risk perception about COVID 19		
Concerns over getting infected with COVID-19	64 (25.0)	34 (27.0)
Concerns over the continuous spread of COVID-19	96 (37.5)	53 (42.1)
Concerns over the new COVID-19 variants	96 (37.5)	39 (30.9)

Table 3 presents levels of quality of life among parents with children 5–11 years old in Malaysia. Almost 30% of the respondents were satisfied with their lives, and 24% were very satisfied with their lives. 24 and 11% of the parents were very dissatisfied and dissatisfied with their lives, respectively. The remaining 11% were neither satisfied nor dissatisfied.

**Table 3:** Level of quality of life among parents with children 5–11 years old.

Overall quality of life ↓	Frequency	Percentage
Very dissatisfied	91	23.82
Dissatisfied	42	10.99
Neither satisfied nor dissatisfied	41	10.73
Satisfied	116	30.37
Very satisfied	92	24.08
Total	382	100

Tables 4, 5 provides the levels of e-health literacy among parents with children 5–11 years old in Malaysia. Above 90% of the respondents agreed to the functional, communicative, critical, and translational criterions of e-health literacy. The mean scores obtained from the respondents’ answers was 4.13 for the functional criteria, 3.87 for the communication criteria, 3.95 and 4.15 for the critical and translational criterions, respectively, which indicated that most of the respondents agreed to the items asked to them.

**Table 4:** e-health literacy among parents with children 5–11 years old.

	Frequency	Percentage
Functional		
Agree	348	91.1
Disagree	34	8.9
Communicative		
Agree	368	96.34
Disagree	14	3.66
Critical		
Agree	366	95.81
Disagree	16	4.19
Translational		
Agree	345	90.31
Disagree	37	9.69

**Table 5:** Levels of e-health literacy among parents with children 5–11 years old in Malaysia.

Transactional e-Health literacy	Score	Mean Score	Description
Functional	4.07	4.13	Can summarize basic health information from the Internet in my own words.
	4.32		Know how to access basic health information on the Internet
	4.08		Can use my computer to create messages that describe my health needs
	4.04		Have the skills I need to tell someone how to find basic health information on the Internet
Communicative	4.14	3.87	Can achieve my health information goals on the Internet while helping other users achieve theirs
	3.76		Have the skills I need to talk about health topics on the Internet with multiple users at the same time
	3.95		Can identify the emotional tone of a health conversation on the Internet
	3.75		Have the skills I need to contribute to health conversations on the Internet
	3.77		Have the skills I need to build personal connections with other Internet users who share health information
Critical	3.96	3.95	Can tell when an Internet user is a credible source of health information
	3.88		Can tell when health information on the Internet is fake
	3.84		Can tell when a health website is safe for sharing my personal health information
	4.06		Can tell when information on the Internet is relevant to my health needs
	3.99		Know how to evaluate the credibility of Internet users who share health information
Translational	4.17	4.15	Can use the Internet to learn how to manage my health in a positive way
	4.15		Can use the Internet as a tool to improve my health
	4		Can use information on the Internet to make an informed decision about my health
	4.27		Can use the Internet to learn about topics that are relevant to me

Tables 6, 7 shows the levels of 5C psychological antecedents of vaccination among parents with children 5–11 years old in Malaysia. The percentages of agreement and disagreement varied across the 5C areas. 73% agreed in the confidence area, while 52% disagreed in the complacency area. 39% of the respondents agreed in the constraints area, while 71 and 74% agreed in the calculation and collective responsibility areas, respectively. As observed in Table 7, the mean score for the set of questions was above 4 for the confidence, calculation and collective responsibility areas.

**Table 6:** 5C Psychological antecedents of vaccination.

	Frequency	Percentage
Confidence		
Agree	279	73.04
Disagree	103	26.96
Complacency		
Agree	182	47.64
Disagree	200	52.36
Constraint		
Agree	149	39.01
Disagree	233	60.99
Calculation		
Agree	283	74.08
Disagree	99	25.92
Collective responsibility		
Agree	270	70.68
Disagree	112	29.32

**Table 7:** Levels of 5C psychological antecedents of vaccination among parents with children 5–11 years old in Malaysia.

5C area	Score	Mean score	Description
Confidence	4.15	4.15	Completely confident that COVID-19 vaccines are safe
	4.17		Completely confident that COVID-19 vaccines are effective
	4.14		Confident that public authorities decide in the best interest of the community
Complacency	3.09	3.31	Vaccination against COVID-19 for children is unnecessary
	3.5		The immune system of children is so strong; it also protects them against COVID-19
	3.33		COVID-19 disease in children is not severe; vaccinate them is superfluous
Constraint	2.84	2.92	Everyday stress prevents me from having my child vaccinated against COVID-19
	3.12		Inconvenient to have my child vaccinated against COVID-19
	2.79		Healthcare facility visits makes me uncomfortable
Calculation	4.09	4.21	I weigh its benefits and risks to make the best decision possible
	4.1		I closely consider whether COVID-19 vaccine is useful for my child
	4.43		It is important for me to fully understand the topic of vaccination before I get my child vaccinated
Collective responsibility	3.92	4.11	Like everyone else, I must get my child vaccinated
	4.15		Having my child vaccinated against COVID-19 can protect people with a weaker immune system
	4.27		Vaccination against COVID-19 is a collective action to prevent the spread of the disease

Table 8 provides the relationship between socio-economic and demographic variables, quality of life, transactional e-health literacy, 5C psychological antecedents to vaccine hesitancy. The left part of the table gives us the association between vaccine hesitancy and the different independent variables using Pearson’s Chi-Square test. The right part of the table shows the univariate and multivariate logistic regression with vaccine hesitancy as the binary outcome variable. The unadjusted model (univariate regression) shows the effect of each independent variable on the dependent variable separately. The adjusted model (multivariate regression) shows the overall effects of all the independent variables when acting together on the dependent variable. From the *p*-values obtained from the Chi-Square tests, gender, employment status, and parent’s COVID-19 vaccination status are significantly associated with vaccine hesitancy among parents (*p* < 0.05). Among transactional e-health literacy, only the communication component had a significant association (*p* < 0.05). Among the 5C psychological antecedents, confidence, calculation and collective responsibility were significantly associated (*p* < 0.05) with vaccine hesitancy.

**Table 8:** Relationship between sociodemographic factors, quality of life, e-health literacy, psychological antecedents to vaccine hesitancy.

Parameters	Hesitancy *n* (%)	*p*-value	Univariate analysis		Multivariate analysis	
			OR (95% CI)	*p*-value	Adjusted OR (95% CI)	*p*-value
Total = 382	126 (33.0)					
Age (mean ± SD)	37.6 ± 11.4	0.076	1.01 (0.99–1.02)	0.54	1.00 (0.97–1.03)	0.984
Gender		0.027				
Male	58 (46.0)		Ref.		Ref.	
Female	68 (54.0)		0.61** (0.4–0.95)	0.028	0.60* (0.34–1.06)	0.077
Marital status		0.061				
Single	25 (19.8)		Ref.		Ref.	
Married	86 (68.3)		2.01*** (1.19–3.39)	0.009	1.45 (0.63–3.32)	0.38
Divorced	8 (6.4)		2.66* (0.95–7.45)	0.063	2.03 (0.53–7.86)	0.303
Widowed	1 (0.8)		1.66 (0.14–19.08)	0.684	0.74 (0.05–1.31)	0.821
Single Parent	6 (4.8)		1.05 (0.38–2.91)	0.928	0.65 (0.19–2.26)	0.498
Highest qualification level		0.171				
Primary	16 (12.7)		Ref.		Ref.	
Secondary	13 (10.3)		2.01 (0.84–4.84)	0.118	8.80*** (2.44–31.79)	0.001
Post-Secondary (Pre University/Diploma)	54 (42.9)		1.94** (1.02–3.71)	0.044	5.21*** (2.10–13.41)	0.000
Tertiary education (Degree/Master)	43 (34.1)		1.99** (1.02–3.88)	0.044	6.77*** (2.25–20.35)	0.001
Household income (Monthly)		0.492				
B40 (equals/less than RM 4,850)	61 (48.4)		Ref.		Ref.	
M40 (RM4,850 to RM10,959)	46 (36.5)		0.92 (0.58–1.47)	0.739	0.77 (0.42–1.42)	0.406
T20 (more than RM10,960)	19 (15.1)		1.39 (0.72–2.69)	0.326	1.55 (0.66–3.59)	0.313
Ethnicity		0.094				
Malay	34 (27)		Ref.		Ref.	
Chinese	28 (22.2)		1.65 (0.89–3.04)	0.11	23.33** (2.07–42.31)	0.011
Indian	59 (46.8)		1.65* (0.99–2.74)	0.055	18.18** (1.85–38.37)	0.013
Others	5 (4.0)		0.68 (0.24–1.95)	0.468	1.94 (0.31–12.16)	0.479
Religion		0.681				
Islam	39 (31.0)		Ref.		Ref.	
Christianity	17 (13.5)		1.42 (0.71–2.87)	0.323	0.11** (0.01–1.00)	0.05
Buddhism	17 (13.5)		1.42 (0.71–2.87)	0.323	0.09* (0.01–1.06)	0.056
Hinduism	49 (38.9)		1.4 (0.84–2.33)	0.196	0.11* (0.01–1.03)	0.053
Others	4 (3.2)		1.01 (0.3–3.4)	0.993	0.06** (0.004–0.93)	0.044
Employment status		0.01				
Employed; full-time	78 (61.9)		Ref.		Ref.	
Employed; part-time	9 (7.1)		1.8 (0.68–4.71)	0.235	1.32 (0.37–4.72)	0.659
Self-employed	12 (9.5)		1.13 (0.52–2.46)	0.751	0.64 (0.24–1.68)	0.366
Retired/Pensioners	3 (2.4)		2.69 (0.44–16.46)	0.284	0.83 (0.09–7.23)	0.864
Housewife/Househusband	10 (7.9)		1.06 (0.46–2.42)	0.898	1.39 (0.49–3.97)	0.533
Unemployed	14 (11.1)		0.36*** (0.19–0.69)	0.002	0.28** (0.10–0.0.75)	0.012
Residential area		0.87				
Rural	27 (21.4)		Ref.		Ref.	
Urban	99 (78.6)		0.96 (0.57–1.61)	0.87	0.80 (0.39–1.65)	0.552
Insurance status of the child		0.482				
Insured	92 (73.0)		Ref.		Ref.	
Not insured	34 (27.0)		0.84 (0.53–1.36)	0.482	0.73 (0.37–1.43)	0.357
Parent’s comorbidity status		0.953				
With chronic illnesses; e.g.: asthma, autoimmune disease, heart disease etc.	18 (14.3)		Ref.		Ref.	
Without chronic illnesses	108 (85.7)		0.98 (0.53–1.81)	0.953	0.70 (0.30–1.64)	0.416
Comorbidity status of your child		0.323				
With chronic illnesses; e.g.: asthma, autoimmune disease, heart disease, etc.	6 (4.8)		Ref.		Ref.	
Without chronic illnesses	120 (95.2)		1.6 (0.62–4.12)	0.327	3.52* (0.84–14.67)	0.084
Parent’s COVID-19 vaccination status		0				
Not vaccinated	0 (0)		Ref.		Ref.	
Completed 1 dose	2 (1.6)		-	-	-	-
Completed 2 doses	35 (27.8)		3.93*** (2.2–7.03)	0	7.26*** (3.23–16.29)	0.000
Completed more than 2 doses	89 (70.6)		-	-	-	-
Oldest child’s (5–11 years) COVID-19 vaccination status		0.164				
Not vaccinated	54 (42.9)		Ref.		Ref.	
Completed 1 dose	23 (18.3)		0.75 (0.41–1.38)	0.359	0.68 (0.32–1.46)	0.326
Completed 2 doses	49 (38.9)		0.63* (0.39–1.02)	0.059	0.28*** (0.13–0.59)	0.001
Side effects experienced by respondent after getting the COVID-19 vaccination		0.943				
Yes	44 (34.9)		Ref.		Ref.	
No	82 (65.1)		1.02 (0.65–1.59)	0.943	0.98 (0.48–1.97)	0.945
Side effects experienced by the oldest child (5–11 years) after getting any vaccination before		0.537				
Yes	25 (19.8)		Ref.		Ref.	
No	101 (80.2)		0.84 (0.49–1.45)	0.538	0.42** (0.18–0.99)	0.049
Risk perception about COVID-19		0.448				
Concerns over getting infected with COVID-19	34 (27.0)		Ref.		Ref.	
Concerns over the continuous spread of COVID-19	53 (42.0)		1.04 (0.61–1.77)	0.888	1.10 (0.55–2.18)	0.795
Concerns over the new COVID-19 variants	39 (31.0)		0.77 (0.44–1.34)	0.346	0.69 (0.33–1.39)	0.3
Overall quality of life: how would you rate your quality of life?		0.635				
Very dissatisfied	30 (23.8)		Ref.		Ref.	
Dissatisfied	13 (10.3)		0.91 (0.42–2)	0.817	0.62 (0.23–1.730)	0.365
Neither satisfied nor dissatisfied	11 (8.7)		0.75 (0.33–1.69)	0.481	0.69 (0.20–2.39)	0.557
Satisfied	36 (28.6)		0.92 (0.51–1.65)	0.767	0.42 (0.15–1.21)	0.11
Very satisfied	36 (28.6)		1.31 (0.71–2.39)	0.386	1.01 (0.32–3.17)	0.98
Transactional e-Health literacy						
Functional		0.067				
Disagree	16 (12.7)		Ref.		Ref.	
Agree	110 (87.3)		0.52 * (0.26–1.06)	0.071	1.07 (0.29–3.87)	0.922
Communicative		0.002				
Disagree	10 (7.9)		Ref.		Ref.	
Agree	116 (92.1)		0.18 ** (0.06–0.6)	0.005	0.30 (0.05–1.86)	0.196
Critical		0.043				
Disagree	9 (7.1)		Ref.		Ref.	
Agree	117 (92.9)		0.37 * (0.13–1.01)	0.051	0.43 (0.09–2.08)	0.297
Translational		0.509				
Disagree	14 (11.1)		Ref.		Ref.	
Agree	112 (88.9)		0.79 (0.39–1.59)	0.51	2.40 (0.67–8.63)	0.18
5C psychological antecedents						
Confidence		0.000				
Disagree	61 (48.4)		Ref.		Ref.	
Agree	65 (51.6)		0.21*** (0.13–0.34)	0.000	0.20*** (0.08–0.46)	0.000
Complacency		0.279				
Disagree	61 (48.4)		Ref.		Ref.	
Agree	65 (51.6)		1.27 (0.83–1.94)	0.279	2.69** (1.20–6.00)	0.02
Constraint		0.632				
Disagree	79 (62.7)		Ref.		Ref.	
Agree	47 (37.3)		0.89 (0.58–1.39)	0.632	1.19 (0.55–2.56)	0.663
Calculation		0.005				
Disagree	44 (34.9)		Ref.		Ref.	
Agree	82 (65.1)		0.51*** (0.32–0.82)	0.005	6.99*** (1.92–25.49)	0.003
Collective responsibility		0.000				
Disagree	67 (53.2)		Ref.		Ref.	
Agree	59 (46.8)		0.19*** (0.12–0.30)	0.000	0.06*** (0.02–0.21)	0.000

*means *p*-value < 0.1, **means *p*-value < 0.05 and ***means *p*-value < 0.01.

Results from the univariate logistic regression suggest that parents who were married were 2 times [OR: 2.01; CI: 1.19–3.39] more likely to hesitate in vaccinating their children than those who were single. Parents with post-secondary and tertiary education also had significant higher risks of hesitancy than those with primary education (*p* < 0.05). Unemployed parents were 36% less likely to report hesitancy [OR: 0.36, CI: 0.19–0.69, *p* = 0.002] than those who were full-time employed. Parents who expressed lower confidence, calculation, and collective responsibility were also less likely to report vaccine hesitancy from the unadjusted regression.

After including all the independent variables together in the adjusted model, we found that parents with secondary [OR: 8.80; CI: 2.44–31.79, *p* = 0.001], post-secondary [OR: 5.21; CI: 2.10–13.41, *p* = 0.000] and tertiary education [OR: 6.77; CI: 2.25–20.35, p = 0.001] were significantly more likely to express vaccine hesitancy than those with primary education. Parents of Chinese and Indian ethnicity were 23 times and 18 times more likely to report hesitation than Malay people, respectively. Unemployed parents were 28% less likely to express hesitancy [OR: 0.36, CI: 0.19–0.69, *p* = 0.012] than those who were full-time employed. Parents who expressed low confidence and collective responsibility were significantly less likely to report vaccine hesitancy than those who express high agreement. However, people low in complacency and calculation criteria had significantly higher likelihood of vaccine hesitancy (3 and 7 times, respectively) than those who agreed. Other independent variables did not have any significant association with vaccine hesitancy as per the multivariate analysis.

## Discussion

The core aims of this study were to explore Malaysian parents’ hesitancy, health literacy, and behaviors surrounding COVID-19 vaccinations of their children 5–11 years old, and to examine the psychological barriers that may prevent parents in Malaysia from getting their children vaccinated against the COVID-19 virus. We found that 32.98% of participants expressed hesitancy in allowing their children 5–11 years old to receive COVID-19 vaccines. This finding was similar to the study investigating the willingness of parents to vaccinate their children under 12 years old in Malaysia, where 26.4% of the participants were either unwilling or hesitant (18). Multiple studies investigating attitudes toward vaccinations among the Malaysian adult population reported varied acceptance rates (i.e., between 27.7 and 96%) (52–55). Parents’ experiences and attitudes toward vaccination may play a role in their decision to vaccinate their children. Compared to other countries, parents from Italy and China showed a similar acceptance rate to vaccinate their children. In contrast, parents expressed greater vaccine hesitancy in countries such as Germany, Turkey, and Saudi Arabia (56–60).

Malaysia is a multiracial, multicultural country. Malays and the aborigine people of the country formed the largest ethnic group (61). Higher hesitancy to vaccinate their children was expressed among Chinese and Indian races. This finding was in line with studies investigating the willingness of the country’s adult population in COVID-19 vaccination (53, 62). According to the SAGE working group report, culture and religion were the determinants of vaccine hesitancy via contextual influences (63). This may explain our finding on the differences in vaccine hesitancy among the ethnic groups. Nevertheless, our study found no significant association between most major religions (Islam, Christianity, Buddhism, and Hinduism) and vaccine hesitancy. This finding opposes results of previous studies in Malaysia in which religion was a significant predictor of vaccine hesitancy (62, 64, 65). Islam is the religion with most adherents in the country as 61.3% of Malaysians are Muslims, followed by Buddhism, Christianity, and Hinduism (66). Larger sample sizes are needed to obtain participants with religions proportionate to the country’s population.

Further, higher educational levels was associated with a higher levels of vaccine hesitancy. This result echoed a previous study conducted in Malaysia, in which parents with higher education were more unwilling to vaccinate their children (18). Similar results were obtained in studies conducted in Saudi Arabia and Turkey (59, 67). It is possible that more educated parents may be more skeptical and perceive that the vaccine is unsafe and ineffective for their children. On the contrary, studies reported that higher parental education was associated with higher acceptance vaccinating children in other regions of the world, including China, Thailand, Italy, and the United States (56–59). The SAGE Working Group observed such diverse educational levels’ effects on vaccine hesitancy. The working group reported that higher education may result in higher or lower vaccine hesitancy, despite better education often resulting in better overall health (63).

We found that only communicative eHealth literacy significantly reduced the risk of vaccine hesitancy, whereas the other dimensions of eHealth literacy were not significantly correlated with parental vaccine hesitancy. Studies in Asian countries show significant associations between eHealth literacy and hesitancy toward COVID-19 vaccination (68–70). However, studies on the impact of eHealth literacy on various health related behaviors and attitude are relatively scarce in Malaysia. Therefore, more research is needed in this area to better understand the detrimental effects of misinformation on important health-related issues such as vaccination (71).

We found that higher confidence from the 5C model reduced the risk of vaccine hesitancy among parents. This finding was similar to the studies in Switzerland and Israel, where confidence predicted intentions to vaccinate children against COVID-19 (51, 72). A Malaysian study conducted among the adult population found significant correlation between confidence and vaccine hesitancy (73). The population from our study supported this and showed significant vaccine hesitancy when expressing complacency. However, two studies conducted in Switzerland and Malaysian did not obtain significant correlation between complacency with vaccine hesitancy (72, 73). Parents expressed high calculation – referring to people undergoing extensive information gathering – were 7 times more likely to be vaccine-hesitant as compared to who were low. Various studies have shown that calculation may predict lower vaccine intention (72–75). Depending on the sources of information search, calculation may influence vaccination attitudes in diverse ways. For example, if anti-vaccination or vaccine-critical information was found, calculation may potentially lead to higher hesitancy (50). Healthcare providers may play an important role in providing correct and valuable information regarding vaccines to parents, which may influence their willingness to vaccinate their children. This has been supported in Italy and China (5, 6).

Parents with higher collective responsibility were found to have lower vaccine hesitancy as people would be willing to receive vaccination for the benefits of others. Collective responsibility was found to be a strong predictor of vaccine acceptance in many studies across the globe (72–74, 76). In our study, the only psychological antecedent in the 5C model that was not associated with negative vaccination attitudes was constraint.

Studies from Switzerland and Bangladesh yielded similar results in the aspect of constraint (72, 75). As opposed to our finding, previous research has found that constraint was a significant predictor of vaccine attitudes (73). All in all, the 5C model of psychological antecedents were important predictors of vaccine attitude in our study population.

This study uses eHealth literacy and the 5C model of psychological antecedents to explore Malaysian parents’ hesitancy, health literacy, and behaviors surrounding COVID-19 vaccinations of their children 5–11 years old. It has been found that parents who are in agreement with confidence and collective responsibility are at a lower risk of vaccine hesitancy in their children, as well as complacent parents, who may express a lower intention to receive the vaccination. The results of this study suggest the need for specific interventions in the future to reduce vaccine hesitancy in our practice as a whole.

A limitation of the TeHLI is that it is a self-reported measure. As such, this assessment was not objective as only the respondent’s perceptions toward their own eHealth literacy competencies are measured. Even so, the TeHLI is an instrument built on a robust theoretical foundation and serves as a useful tool for the quick assessment of eHealth literacy levels. Further, the cross sectional design of the study is another important limitation as parental willingness to vaccinate their children is a dynamic process and the picture captured in the current study might have difficulty to reflect the changes over time. Selection bias as a result from convenience sampling method employed in this study may potentially affect the findings. The survey was conducted via online platform and this could also lead to selection bias as eHealth literacy of the people who commonly use the internet and social media may differ from those who are not. This could affect the generalizability of the result from this study. Social desirability bias may be present in the study as all the instruments used were of a self-reporting nature, therefore participants may underreport behaviors or ideas that might be deemed as unfavorable by others.

In conclusion, nearly a third of the participants in the study were hesitant to vaccinate their children. This study also suggests that highly educated parents are more skeptical and more likely to perceive the vaccine as unsafe and ineffective for their children. It is therefore critical to disseminate the required information about the vaccine safety to educated groups. Further, the 5C model of psychological antecedents explained probable reasons behind parental vaccine hesitancy in Malaysia. This is the first study that has examined vaccine hesitancy for children from their parents’ perspective in Malaysia, and the findings could be used by policymakers to understand the ways through which they can improve the vaccination for children. In addition, the study should be replicated, and the results contrasted with countries from Europe (e.g., Italy, Austria and Germany), who faced long lockdowns and conducted studies regarding vaccine hesitancy.

## Data availability statement

The original contributions presented in the study are included in the article/supplementary material, further inquiries can be directed to the corresponding authors.

## Ethical statement

The studies involving human participants were reviewed and approved by Ethics approval was obtained from the Medical Research and Ethics Committee of the Management and Science University Shah Alam, Selangor, Malaysia. The patients/participants provided their written informed consent to participate in this study.

## Author contributions

All authors have made a substantial, direct, and intellectual contribution to the work, and each author has reviewed and approved the final manuscript for publication.

## Conflict of interest

The authors declare that the research was conducted in the absence of any commercial or financial relationships that could be construed as a potential conflict of interest.

## Publisher’s note

All claims expressed in this article are solely those of the authors and do not necessarily represent those of their affiliated organizations, or those of the publisher, the editors and the reviewers. Any product that may be evaluated in this article, or claim that may be made by its manufacturer, is not guaranteed or endorsed by the publisher.
